# Editorial: From Traditional to Modern: Progress of Molds and Yeasts in Fermented-Food Production

**DOI:** 10.3389/fmicb.2022.876872

**Published:** 2022-03-17

**Authors:** Wanping Chen, Xucong Lv, Van-Tuan Tran, Jun-ichi Maruyama, Kap-Hoon Han, Jae-Hyuk Yu

**Affiliations:** ^1^Department of Molecular Microbiology and Genetics, Georg-August-Universität Göttingen, Göttingen, Germany; ^2^College of Biological Science and Technology, Fuzhou University, Fuzhou, China; ^3^Department of Microbiology & National Key Laboratory of Enzyme and Protein Technology, University of Science, Vietnam National University, Hanoi, Vietnam; ^4^Department of Biotechnology, The University of Tokyo, Tokyo, Japan; ^5^Department of Pharmaceutical Engineering, Woosuk University, Wanju, South Korea; ^6^Department of Bacteriology, University of Wisconsin-Madison, Madison, WI, United States; ^7^Department of Systems Biotechnology, Konkuk Institute of Science and Technology, Konkuk University, Seoul, South Korea

**Keywords:** fermented food, mold, yeast, *Aspergillus*, *Saccharomyces*, Koji

## Introduction

Molds (filamentous fungi) and yeasts have been used for the production of foods and beverages throughout the world since ancient times (Venturini Copetti, 2019). For example, yeasts widely contribute to various alcoholic fermentations, such as beer and wine, and non-alcoholic fermentations, such as bread and coffee (Maicas, 2020; Iorizzo et al., 2021). In western countries, *Penicillium* spp. are used for ripening cheeses and meats (Chávez et al., 2011). In the Orient, there are also a variety of fermented foods produced by molds and/or yeasts, which have profoundly shaped the eating habits of the locals. For example, *Aspergillus oryzae* is widely applied for brewing soy sauce, douche, miso, sake, and doenjang etc. (Hong and Kim, 2020; Daba et al., 2021).

Despite the great economic, cultural, and social values of traditional fungal fermentations in food production, these have strong regional characteristics. Therefore, this Research Topic tried to offer a collection of articles associated with different types of fermentation products and processes from different regions, which could provide a global perspective for molds and yeasts in fermented food production. Based on the research content, the articles in this collection could be divided into the following sections.

## Characterization and Improvement of Fermentation Process

Tan et al. optimized the production of the soursop kombucha by response surface method, and determined the effects of different storage conditions on the quality, metabolites, and biological activity. This study revealed that prolonged storage conditions have a high potential to improve the quality, metabolites content, biological activity, and the Halal status of the soursop kombucha.

Liu et al. evaluated the effect of different starter cultures on the ripening of dry fermented sausages and found that the lactic acid bacteria could rapidly reduce the pH value of the products and inhibit *Enterobacter putrefaciens* to ensure safety, while the yeasts contributed more in flavor formation and effective inhibition of lipid oxidation.

Xiao et al. investigated the effect of three yeasts on the Kazak cheese quality and flavor, and the results showed that the texture of cheese added with yeasts was more brittle, suggesting that yeasts are important auxiliary starters for cheese production.

Lee et al. used *Aspergillus cristatus* for the liquid-state and solid-state fermentation of *Panax notoginseng* and examined the contents of protopanaxadiol and protopanaxatriol representing antioxidant activity and skin anti-aging. The results suggested that fermentation of *P. notoginseng* by *A. cristatus* could enhance the quality and availability of bioactive compounds associated with skin anti-aging.

Qin et al. studied the contribution of four non-*Saccharomyces* yeast strains *Issatchenkia terricola* SLY-4, *Pichia kudriavzevii* F2-24, *P. kudriavzevii* F2-16, and *Metschnikowia pulcherrima* HX-13 with β-glucosidase activity to the flavor and quality of wine making. The results showed that in general, the sensory evaluation score of adding non-*Saccharomyces* yeast-fermented wine was better than that of *Saccharomyces cerevisiae*.

Fan et al. isolated a *Clavispora lusitaniae* strain capable of producing a large amount of ethyl caproate from Daqu, a crude fermentation starter for Baijiu, and optimized the fermentation conditions for ethyl caproate production. The results also revealed that this strain can produce many flavor compounds important for high-quality Baijiu and has potential applications in improving the flavor and quality of Baijiu.

Yuan and Chen designed a double-sided petri dish to characterize the cocultivation of *Monascus* spp. and *Aspergillus niger* inspired by black-skin-red-koji. The results indicate that the designed petri dish might be an efficient tool for the investigation of microbial interaction in the laboratory.

Farh et al. cultured *Saccharomyces fibuligera* strains used for the production of *makgeolli* (Korean rice wine) under different pH conditions, and investigated the effect on their enzyme production and gene expression. The results showed that the decrease in pH induced a dimorphic lifestyle switch from yeast cell formation to hyphal growth in *S. fibuligera* and caused a decrease in carbohydrate hydrolyzing enzyme production, and marked changes in the expression of genes related to enzyme production and pH adaptation.

Yang et al. investigated the effects of *Flos sophorae immaturus* on the stability of *Monascus* pigments, the flavor profiles, and the sensory characteristics of Hongqu Huangjiu. The study suggested that the addition of *Flos sophorae immaturus* could be a new strategy for improving the stability of photosensitive pigments and adjusting the aroma of Hongqu Huangjiu.

Chen et al. prepared an artificial starter culture by using the core microbial species of JIUYAO to produce Chinese rice wine and compared its fermentation activity and flavor profiles with traditional JIUYAO and a commercial starter culture. The results showed that the fermentation activity and flavor profiles of the artificial starter resembled those of traditional JIUYAO.

## Microbiota of Qu and Fermentation Process

The microbiota of fermented meat products is closely linked with their characteristics (Van Reckem et al., 2019). Chen et al. studied the microbial community structure of Mianning ham and found that *Penicillium lanosum, Penicillium nalgiovense, Debaryomyces hansenii, Staphylococcus equorum*, and *Erwinia tasmaniensis* were isolated from the surfaces of the hams by the traditional culture method, while *Aspergillus, Penicillium*, and *Wallemia* were the dominant genera by Illumina high-throughput sequencing. Moreover, the authors identified a total of 60 flavor substances in the hams. Wang et al. summarized and compared the microbial diversity of Chinese ham, sausage, preserved meat, pressed salted duck, preserved fish, and air-dried meat, which is a useful review for the microbial compositions of fermented meat products in China. Wang et al. studied the correlation between the microbial communities and volatile flavor compounds of 15 Suan zuo rou (a traditional fermented pork product) samples from three regions in Guizhou province, and revealed that *Brochothrix, Candida, Debaryomyces, Kazachstania, Lactobacillus, Leuconostoc, Pediococcus, Pichia, Staphylococcus, Weissella*, and *Wickerhamomyces* were highly correlated with 48 volatile flavor compounds.

Qu (Koji) is usually composed of cooked grains inoculated with a fermentation culture and used as the fermentation starter in the production of many Oriental fermented foods (Zhu and Tramper, 2013). The investigation of Qu microbiota could contribute to the understanding on how the fermentation starts. Zhao et al. analyzed the microbiota of three typical traditional Xiaoqu from the Guizhou province in China by metagenomic sequencing, and revealed that *Lactobacillus, Bacillus, Acinetobacter, Leuconostoc*, and *Weissella* were the dominant bacterial genera, while *Aspergillus, Saccharomyces, Pichia, Rhizopus*, and *Phycomyces* are the predominant fungal genera. Zhang et al. explored the microbial shifts in high-temperature Daqu during maturation, and revealed that the predominant bacteria shifted from *Saccharopolyspora* (outer) and *Staphylococcus* (inner) to *Kroppenstedtia* (both outer and inner), while the predominant fungi shifted from *Thermoascus* (both outer and inner) to *Byssochlamys* (outer) and *Fusarium* (inner).

Baijiu is a good representative to study the succession of complex microbiota during fermentation. Hao et al. analyzed the microbial community structure in the initial fermentation of Maotai-flavor Baijiu by high-throughput sequencing and found that *Lactobacillus, Pichia*, and *Saccharomyces* were the dominant microorganisms in the initial fermentation. It also suggested that reducing sugar was the key driving factor for microbial succession in the heap fermentation, while acidity, alcohol, and temperature were the main driving forces in pit fermentation. Hu et al. tracked the changes of microbial community in the production of rice-flavor Baijiu by high-throughput sequencing technology and revealed that the dominant bacteria were *Lactobacillus*, while the core fungi were *Saccharomyces* and *Rhizopus*. It inferred that compared to other flavor types of Baijiu, the fewer microbial species but prominent microorganisms may be the main reason for the small variety of flavor substances in rice-flavor Baijiu. Kang et al. investigated the shifts in microbial community diversity of Fuyu-flavor Baijiu from the pretreatment of raw materials to the end of saccharification by high-throughput sequencing and revealed that *Lactobacillus, Weissella*, and *Bacillus* in the bacterial community and *Rhizopus, Candida, Pichia*, and *Aspergillus* in the fungal community are predominant during raw material pretreatment and saccharification processes. It further indicated that during the saccharification process, the cooling grains and rice husks were the main contributors to the bacterial community composition, and Qu was the main contributor to the shaping of the fungal community structure.

In addition, Zhu et al. investigated the succession of fungal communities on Cabernet Sauvignon grapes from an organic vineyard in Xinjiang at different developmental stages and found that *Aspergillus, Malassezia, Metschnikowia*, and *Udeniomyces* were predominant during the unripe stage, whereas *Cladosporium, Cryptococcus, Erysiphe*, and *Vishniacozyma* were dominant in the ripe stages. Ma et al. investigated the dynamic changes of the microbial population and volatile compounds during the spontaneous fermentation of *Petit Manseng* sweet wine and found that *Candida* and *Mortierella* were dominant genera in the fermentation and contributed to the formation of fermentative aroma compounds.

## Functional Components Produced by Fermentation Fungi

Cui et al. deleted or overexpressed 2,3-butanediol dehydrogenase (BDH) coding genes BDH1 and BDH2 to evaluate the effect on the content of acetoin and 2,3,5,6-tetramethylpyrazine in *S. cerevisiae*. This work provides a novel method to improve the quality and beneficial health attributes of Baijiu by increasing 2,3,5,6-tetramethylpyrazine production in *S. cerevisiae* by genetic engineering.

Dai et al. isolated and characterized an aromatic strain of *Zygosaccharomyces rouxi* from the chili sauce. They identified the aromatic alcohol with a rose honey scent as 2-phenylethanol and inferred its possible biosynthetic pathway.

Zhang et al. compared the fermentation broths of *Monascus purpureus* with and without glutamic acid supplementation using a metabolomic profiling approach to identify key metabolites and metabolic pathways for improving the yield of monacolin K. The results suggested that the citric acid cycle is closely related with monacolin K yield.

Husakova et al. tested the activity of red yeast rice extract on germination of *Clostridium* and *Bacillus* spores. The results revealed that the extract added to the medium at a concentration of 2% v/v could fully suppress *Clostridium beijerinckii* spore germination, while the addition of 4% v/v extract to the medium containing 1.3% w/w NaCl could fully inhibit *Bacillus subtilis* spore germination.

Wu et al. investigated the difference in the composition of *Monascus* azaphilone pigments between functional Qu and coloring Qu and analyzed their relationships with antioxidant activity. The results indicated that the seven *Monascus* yellow pigments may be the key active components for coloring Qu to have a more potent antioxidant capacity than functional Qu.

Jiang et al. summarized the types, structures, and biosynthetic pathways of glycosphingolipids in filamentous fungi, and the roles of glycosphingolipids in fungal growth, spore formation, and environmental stress response. Furthermore, this review proposed the advantage, potential development, and application of glucosylceramides and galactosylceramides from filamentous fungi *Aspergillus* spp. in health foods and cosmetics.

## Evaluation and Safety Control of Fermented Products

Zhao et al. explored the flavor characteristics of three kinds of Japanese soy sauce and identified a total of 173 volatile flavor substances and 160 taste compounds. The results revealed that alcohols and aldehydes were in high abundance in Japanese soy sauce, but pyrazines and esters were only a small portion.

Xu et al. summarized the origin, evolution, and control technology of undesirable metabolites (e.g., ochratoxin A, ethyl carbamate, and biogenic amines) in wine. This review also highlighted current wine industry practices of minimizing the number of biotoxins in wine.

Yang et al. summarized the aspects that may affect the formation of Huangjiu flavor compounds. This review also discussed the selection of appropriate raw materials and the improvement of fermentation technologies to promote the flavor quality of Huangjiu. In addition, this review investigated the effects of microbial community composition, metabolic function of predominant microorganisms, and dynamics of microbial community on the flavor quality of Huangjiu.

## Genetic Research of Fermentation Fungi

Jin et al. reviewed the advances in basic research and genetic engineering technologies of the fermentation strain *A. oryzae*, which could open up more effective ways and research space for the breeding of *A. oryzae* production strains in the future. The review of Tanaka and Gomi presented the current knowledge on the regulation of hydrolase gene expression, including carbon catabolite repression, in *A. oryzae*.

Yang et al. studied the role of three kinases, Elm1, Tos3, and Sak1, in the maltose metabolism of baker's yeast in lean dough. The results, for the first time, revealed that Elm1, rather than Tos3 and Sak1, might be the dominant regulator in the maltose metabolism of baker's yeast.

Liu et al. predicted the boundary of the biosynthetic gene cluster of *Monascus* azaphilone pigments (MonAzPs) in *Monascus ruber* M7 by a combination of computational and transcriptional analyses. Then, gene knockouts and analysis of MonAzPs production of the mutants were used to validate the prediction, revealing that the biosynthetic gene cluster consists of 16 genes.

## Phylogenetic, Physiological, and Genomic Features of Fermentation Species

He et al. isolated a *Monascus sanguineus* strain, and speculated that this strain may be a natural nothospecies based on the morphological characteristics and the phylogenetic relationship of *Monascus* species. This study provides new insights into how *Monascus* evolved. Zhou et al. found that *Monascus* has good tolerance to lactic acid and ethanol, while the microbial community was repressed at the condition, and then designed a novel restrictive medium for *Monascus* enrichment from Hongqu based on the synergistic stress of lactic acid and ethanol. This work has great application value for the isolation of *Monascus* strain from Hongqu and the better development of its germplasm resources.

Han et al. performed a population genomic analysis of massive *S. cerevisiae* isolates, and suggested that the wild and domesticated populations of *S. cerevisiae* are separated and the domesticated population diverges into two distinct groups associated with solid- and liquid-state fermentations from a single ancestor. This study improves the understanding on the genetic diversity of *S. cerevisiae* strains and how they evolved.

Chacón-Vargas et al. performed a population genomic analysis of *A. oryzae* isolates, especially focusing on the comparison of industrial strains *A. oryzae* 14160 and RIB 40, and revealed substantial genome and phenotypic variation especially for alpha-amylase genes within *A. oryzae*. This study provides insights into the adaptive evolution of *A. oryzae* during domestication.

## Opinions, Perspectives, and Reviews on Fungal Fermentation Industry and Research

Red mold rice is the fermentation product of *Monascus* spp. and widely used as a food colorant, brewing starter, and monacolin K supplement (Chen et al., 2015). In the opinion of Wang et al., it summarized the current research themes on *Monascus* and proposed some future issues on red mold rice production, the correlation of *Monascus* polyketide biosynthetic pathways and their regulation, and the relationship between *Monascus* development and secondary metabolism. In the perspective of Yanli and Xiang, it summarized the bioactive components of functional red mold rice (FRMR) and their functions, and proposed the efficient strategies for FRMR production, and future directions and challenges of FRMR application.

Sake is a Japanese traditional fermented alcoholic drink, which is brewed using koji mold *A. oryzae* to convert the starch in rice into sugar, and sake yeast *S. cerevisiae* further convert into ethanol (Zhang et al., 2020). In the opinion of Nishida, it highlights the bacterial roles in Sake that is considered exclusively mold/yeast-based. In addition, the opinion points out that bacteria should also be considered for the complete safety assessment of Sake.

In the perspective of Carrau and Henschke, it explained the concept of “friendly” yeasts for developing wine starters that do not suppress desirable native microbial flora at the initial steps of fermentation, summarized the roles of non-Saccharomyces yeasts, and proposed that inoculation of *Hanseniaspora vineae* strains could develop ideal conditions for flavor expression of the microbial terroir without the risk of undesirable strains.

Cheese is an ancient traditional fresh or fermented dairy product but with a complex microbial community structure, diverse processing technologies, and flavors. The review of Zheng et al. summarized the research progress on the general processing technology and key control points of natural cheese, the biochemical pathways of cheese flavor formation, the diversity, and the role of yeasts in cheese. This review provides important advances in understanding the effects of different cheese-making techniques and microbial diversity on cheese flavor and quality.

Molds and yeasts play an irreplaceable role in the formation of flavor substances and the production of functional components in traditional Chinese fermented foods. The review of Yang et al. summarized the research progress of molds and yeasts in traditional Chinese fermented foods, including the diversity, population structure and interaction of molds and yeasts, and discussed their application development prospects in related industries.

## Conclusions and Perspectives

**Figure 1:**
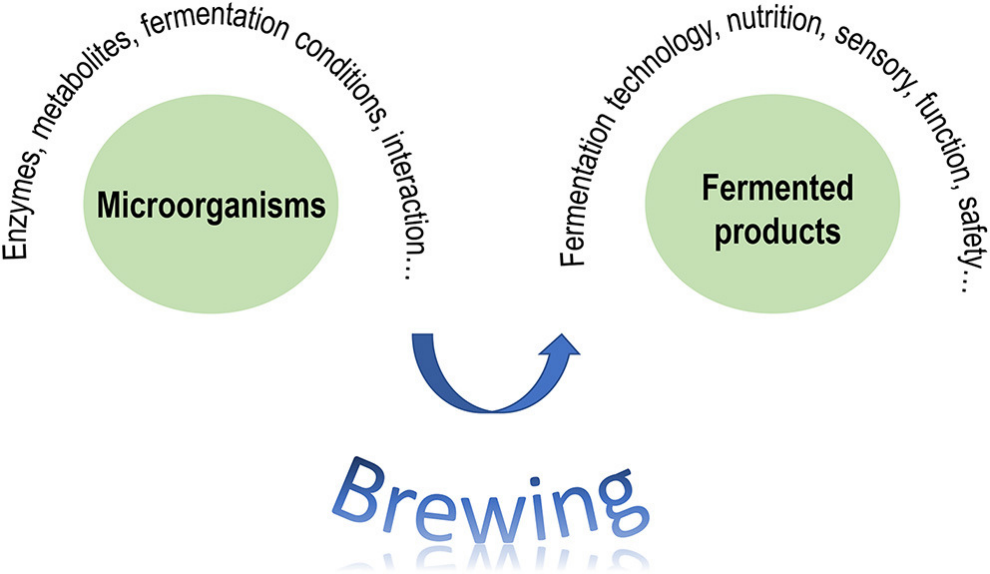
The main research aspects on food fermentation.

In general, the research mainly includes two aspects (Figure 1). The first aspect revolves around fermentation microorganisms to address their enzymes, metabolites, fermentation conditions, interaction, dynamic changes, etc. The other aspect centers on fermented products to study their fermentation technology, nutrition, sensory, safety, etc. The main purpose is to connect the behaviors of fermentation microorganisms and the properties of fermented products.

Although this Research Topic focused on molds and yeasts, the brewing processes from microorganisms to products in most cases are complex and involve a plethora of strains including bacteria. For example, as revealed by many studies in the topic, the microbiota of Qu and Baijiu fermentation process cooperatively involved a high number of fungi and bacteria. In the previous studies, most of research attention has been focused on some key microorganisms. However, as proposed in the opinion of Nishida and perspective of Carrau and Henschke, more attention may need to be paid to the synergy of the microbiome other than several key microorganisms. Hopefully, with the development of new technologies, especially the emerging omics tools like metagenomics, metatranscriptomics, metaproteomics, and microbiomics, further studies will provide a more comprehensive and vivid perspective on food fermentation.

## Author Contributions

WC drafted the manuscript. All authors contributed to the final version and approved it for publication.

## Conflict of Interest

The authors declare that the research was conducted in the absence of any commercial or financial relationships that could be construed as a potential conflict of interest.

## Publisher's Note

All claims expressed in this article are solely those of the authors and do not necessarily represent those of their affiliated organizations, or those of the publisher, the editors and the reviewers. Any product that may be evaluated in this article, or claim that may be made by its manufacturer, is not guaranteed or endorsed by the publisher.
